# Genetic effect of basal metabolic rate on the benign neoplasm of bone and articular cartilage: a Mendelian randomization study

**DOI:** 10.3389/fonc.2024.1446310

**Published:** 2024-09-26

**Authors:** Guijin Huang, Ying Yao, Lin Fan, Sisi Li

**Affiliations:** ^1^ Department of Anesthesiology, Stomatological Hospital of Chongqing Medical University, Chongqing, China; ^2^ Chongqing Key Laboratory of Oral Diseases and Biomedical Sciences, Chongqing, China; ^3^ Chongqing Municipal Key Laboratory of Oral Biomedical Engineering of Higher Education, Chongqing, China

**Keywords:** basal metabolic rate, benign neoplasm, bone, articular cartilage, Mendelian randomization

## Abstract

**Background:**

Previous studies have found an association between basal metabolic rate (BMR) and various malignant neoplasms, including bone tumors. BMR is also associated with bone mineral density, but the causality between BMR and benign neoplasms of bone and articular cartilage remains uncertain.

**Design:**

Single nucleotide polymorphisms (SNPs) associated with BMR (p < 5 × 10^-8^) were used as instrumental variables for Mendelian randomization analysis of neoplasm risk. The inverse variance weighted (IVW) method was the primary approach, with the weighted median and MR-Egger regression serving as supplements.

**Results:**

In this MR analysis, the IVW method supported a causal relationship between BMR and benign neoplasms of bone and articular cartilage (OR = 1.417; 95% CI, 1.039 to 1.930; p = 0.027). No evidence of heterogeneity or pleiotropy in the selected SNPs was found in our study. Thus, based on these results, we discovered a possible causal relationship between BMR and benign neoplasms of bone and articular cartilage.

**Conclusions:**

In this MR study, evidence suggested a genetic correlation between genetically predicted BMR and the risk of neoplasms in bone and articular cartilage.

## 1 Introduction

Basal metabolic rate (BMR) is an indicator of overall body metabolism and may be a proxy for the impact of a specific metabolic profile on cancer risk. Some researchers have found an association between BMR and cancer, including malignant bone neoplasms (1). However, observational studies were susceptible to confounding factors, which may compromise their internal validity. Mendelian randomization (2) studies have investigated and confirmed the effect of BMR on bone cancer (3). Additionally, other studies have shown associations between BMR and bone mineral density, as well as bone regeneration (4–7). One study demonstrated that BMR is positively correlated with bone mineral density in middle-aged to older women (8). Another study found a strong correlation between BMR and femur bone mineral density in men with traumatic spinal cord injury (5). Furthermore, postmenopausal women with higher BMRs tend to have higher bone mineral density (6). These findings suggest that BMR plays a significant role in bone health and regeneration. Nonetheless, the relationship between BMR and benign neoplasms of bone and articular cartilage remains unclear.

Benign bone tumors are a diverse group of non-cancerous growths that arise in bone and cartilage tissues, commonly affecting children and young adults (9). Diagnosis primarily relies on imaging, with biopsy rarely being necessary. The most common types of benign bone tumors include osteochondromas (accounting for 20% to 35% of all benign bone tumors), which account for 20% to 35% of all benign bone tumors, enchondromas, and osteoid osteomas (10). Management varies based on tumor type, location, symptoms, and risk of recurrence. While some tumors are asymptomatic and require no intervention, others may necessitate percutaneous ablation or surgery due to symptoms. Although malignant transformation is rare for benign bone tumors, patients should undergo serial imaging for monitoring.

Currently, the true causal relationship between BMR and benign neoplasms of bone and articular cartilage remains debatable. Methodological limitations of observational studies, such as potential fallacies and confounding, continue to be a concern. Therefore, investigating this relationship using suitable methodology and a large sample size is imperative. MR analysis, which employs genetic variants as instrumental variables (IVs) to determine causal relationships, represents a widely used alternative approach (11–13). MR can ascertain causal effects and account for residual confounding in the presence of inconsistent observational evidence. However, there is a limited number of MR studies on this topic.

In the current study, we conducted an MR analysis to investigate the effect of BMR on benign neoplasms of bone and articular cartilage using data from the UK Biobank and Finngen database. Using a two-sample MR analysis, we aimed to examine the genetic and causal link between basal metabolic rate and benign neoplasms of bone and articular cartilage.

## 2 Materials and methods

### 2.1 Data sources

#### 2.1.1 Overall data declaration

The summarized data used in this study were sourced from a publicly accessible database that compiles genome-wide association study (GWAS) summary data from published studies or GWAS cohorts initiated by reputable and qualified consortiums. All these investigations received ethical approval from their respective institutions’ review committees. To estimate a causal effect of BMR on benign neoplasms of bone and articular cartilage, only summary data were utilized to conduct a two-sample MR study for the current investigation. No additional corrections or adjustments were required.

#### 2.1.2 Study exposure: BMR

The GWAS summarized data for BMR were obtained from the UK Biobank (UKB) through the website https://gwas.mrcieu.ac.uk/datasets/ukb-b-16446/. In our study, European ancestry populations were used among BMR patients, comprising 23,105 cases and 431,769 control participants. To ensure that IVs were strongly associated with BMR, we selected SNPs that showed genome-wide significance (p < 5 × 10^−8^) in their association with BMR as IVs for genetic variants.

#### 2.1.3 Study outcome: benign neoplasm of bone and articular cartilage

Data on benign neoplasm of bone and articular cartilage were extracted from the Finngen database, recruiting a total of 1,190 cases and 178,632 controls. Importantly, these datasets excluded individuals with any form of cancer to control for confounding factors. Confounding factors considered: Age and Sex: These are primary demographic variables known to influence the incidence of neoplasms. Smoking Status: Smoking is a well-known risk factor for various neoplasms, including bone-related conditions. Alcohol Consumption: Alcohol use can influence metabolic rate and has been associated with cancer risk. BMI: BMI is related to both metabolic rate and cancer risk. Physical Activity Level: Physical activity impacts metabolic rate and overall health. Genetic Ancestry: To control for population stratification, we ensured that the genetic data used were from individuals of European ancestry. Comorbidities: Conditions such as diabetes, hypertension, and cardiovascular diseases that can influence metabolic rate and cancer risk were considered. We utilized the PhenoScanner database to ensure that the SNPs selected as instrumental variables were not associated with these confounding factors at genome-wide significance level (p < 5 × 10^−8^). This process ensured that the SNPs used in our MR analysis were not linked to any of the identified confounders, thereby strengthening the validity of our causal inference.

### 2.2 The selection of associated SNPs

To estimate this causal effect, we selected genetic variants as IVs based on the following hypotheses: (1) they must be predictive of BMR, (2) they must be uncorrelated with confounding factors, and (14) they must not influence the benign neoplasm of bone and articular cartilage through any pathway other than BMR. These principles must be adhered to rigorously. Accordingly, we selected BMR-associated SNPs with genome-wide significance (p < 5 × 10^−8^) from the corresponding datasets. We then trimmed for linkage disequilibrium based on the European population (r² < 0.001; distance > 10000 kb) to ensure the independence of the IVs. Next, the PhenoScanner database (http://www.phenoscanner.medschl.cam.ac.uk/) was utilized to verify all selected SNPs and examine potential violations of hypotheses (2) and (14, 15). To minimize bias in the eventual outcomes, SNPs associated with multiple confounding traits at the genome-wide significance level were stringently excluded. We then searched for these remaining SNPs in GWAS datasets on benign neoplasm of bone and articular cartilage, removing any SNPs absent in the database for benign neoplasm of bone and articular cartilage. For any absent SNPs, proxies with a high correlation (r² > 0.8) were selected (palindromes allowed, MAF threshold > 0.3). Additionally, any SNPs strongly associated with benign neoplasm of bone and articular cartilage (p < 5 × 10^−8^) was excluded. Next, we extracted information for every SNPs and calculated the F statistic to reject weak instrumental variables (F < 10), as typically recommended in MR analyses. The formulas are as follows: F = (N-κ-1)R²/[κ(1-R²)], where N represents the total sample size, κ represents the number of IVs, and R² represents the proportion of variation in BMR for each SNP; R² = β²2MAF(1-MAF), where β represents the effect size of SNPs on BMR, and MAF (minor allele frequency) is normally equal to EAF (effect allele frequency) when calculating R² (16, 17). Ultimately, we carefully checked the orientation of genetic effects and eliminated any SNPs by using the Steiger filtering test, applying it to each SNPs and every MR method simultaneously (Steiger p-value > 0.05) (18).

### 2.3 MR statistical analysis

**Figure 1:**
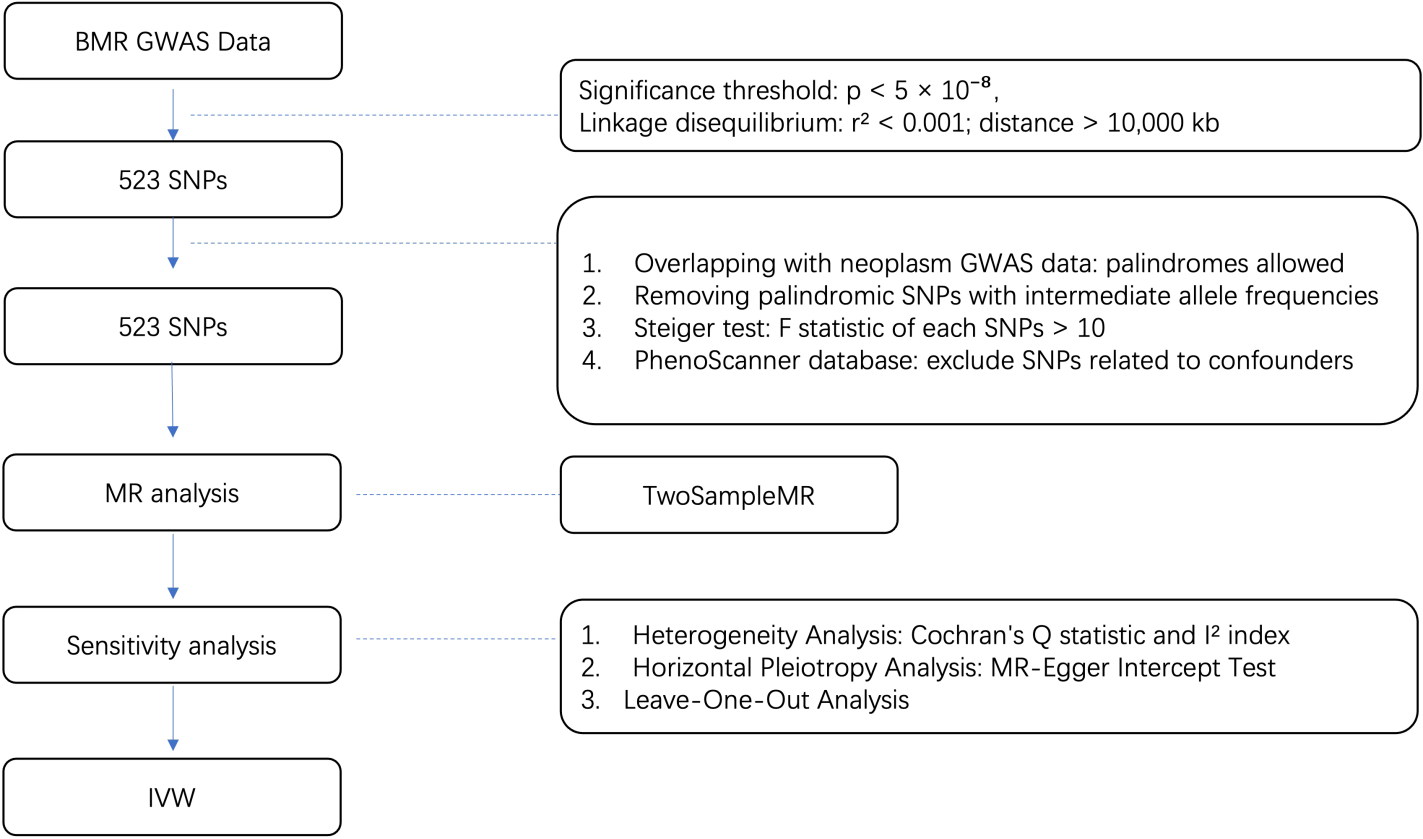
Flow diagram for quality control of the IVs and the whole MR analysis. GWAS, genome-wide association study; SNPs, single-nucleotide polymorphisms; BMR, basal metabolic rate; MAF, Minor allele frequency; EAF, effect allele frequency.

All our MR analyses were performed in the R programming language, version 4.2.3 mainly using the R package “Two Sample MR” (version 0.5.7) and “Mendelian Randomization” (version 0.9.0). Other R packages were also utilized to assist in for formal MR analysis. The whole process is shown in **Figure 1**.

Given the paucity of GWAS data at an individual level, the MR method newly developed in recent years, analyzing with the summary-level data, was applied to assess whether BMR was associated with the benign neoplasm of bone and articular cartilage in a genetically causal relationship. By minimizing the confounding effect of confounding factors and eliminating reverse causation in conventional observational research, MR analysis can reinforce deductions regarding the causative character of exposure-outcome associations as a powerful genetic approach (19). Because genetic alleles linked to exposure are conceptually categorized randomly and are less affected by illness, they are also less likely to be influenced by confounders such as lifestyle and environment. In this study, a systematic investigation of potential confounders was conducted to enhance the validity of our causal inference. Specifically, we considered confounders such as age and sex, which are primary demographic variables influencing the incidence of neoplasms; smoking status, a well-known risk factor for various neoplasms, including bone-related conditions; and alcohol consumption, which can affect metabolic rate and is associated with cancer risk. Additionally, BMI (Body Mass Index), physical activity level, genetic ancestry, and comorbidities such as diabetes, hypertension, and cardiovascular diseases were accounted for, given their influence on metabolic rate and cancer risk. By rigorously addressing these factors, we aimed to minimize confounding and strengthen the robustness of our findings. However, the exposure-outcome pathway may not be unique, which can violate the MR assumption and result in biased causal estimates. To correct for potential horizontal pleiotropy, we employed IVW, MR-Egger, MR-PRESSO and weighted median analytic approaches. The IVW approach is the most widely used MR statistical method. The MR-Egger method allows for horizontal pleiotropy in the included instrumental SNPs, but it is usually not capable of distinguishing between pleiotropy and a causal effect (19). Moreover, the MR-Egger method is susceptible to “weak instrument bias” when it fails to meet the “Inside” and “Nome” assumptions (20). The MR-PRESSO method (Mendelian Randomization Pleiotropy Residual Sum and Outlier) detects and corrects for pleiotropy and identifies and adjusts the outlier SNPs to reduce bias due to pleiotropy. The weighted median method is complementary to MR-Egger, which provides an accurate estimate if no less than half of the genetic information derives from valid SNPs. Each of the three approaches is established on different statistical models (21). The main distinction between the IVW method and MR-Egger regression lies in whether they incorporate an “intercept” term in their respective calculation formulas, often resulting in divergent outcomes. Generally, such inconsistencies between IVW and MR-Egger results primarily stem from limitations in the statistical method and the lower power of MR-Egger (19). Therefore, the IVW method (without an “intercept”) outperforms MR-Egger in scenarios lacking heterogeneity and pleiotropy, owing to its purely linear relationship that passes through the origin (19, 22).

## 3 Results

### 3.1 Genetic instrumental variables for BMR

In the corresponding GWAS database, under the conditions of genome-wide significance level *p*<5 × 10^-8^, R^2 =^ 0.001, and clump=10,000kb, a total of 523 SNPs related to BMR at the genome-wide significance level (*p*<5 × 10^-8)^ were identified. After examining the secondary phenotypes of these SNPs, none of them were significantly associated with confounding traits at the genome-wide level (*p*<5 × 10^−8^). All 523 SNPs met the criteria, with an F-statistic greater than 10 and a Steiger *p*-value less than 0.05.

### 3.2 MR analysis for benign neoplasm of bone and articular cartilage

**Figure 2:**
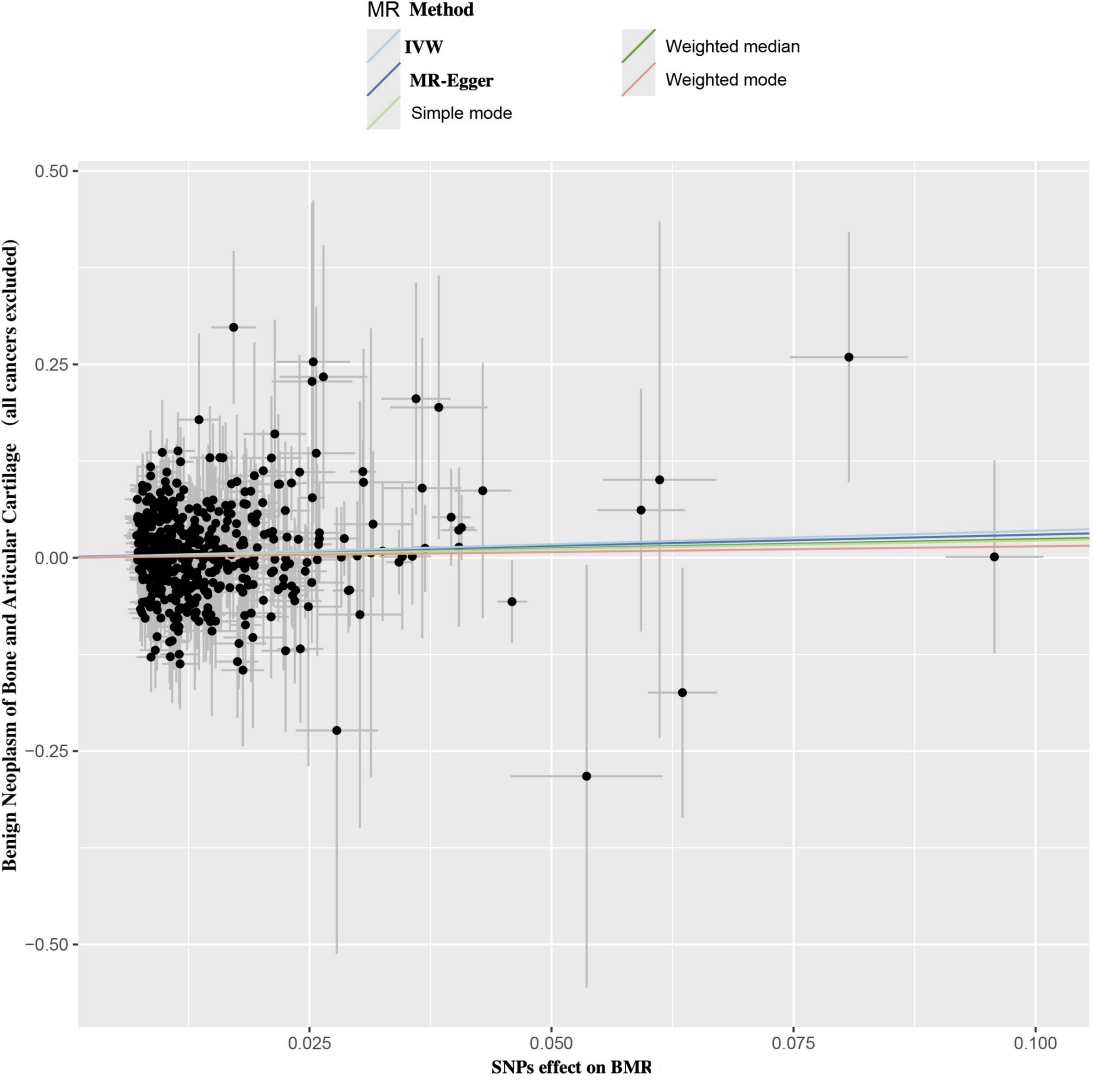
Scatter plot of SNPs relevant to BMR and the risk of benign neoplasm of bone and articular cartilage. Each splash displayed the effect sizes for SNPs-BMR relation (x-axis, SD units) and the SNPs-benign neoplasm of bone and articular cartilage relation (y-axis, log (OR)) with 95% CIs. Using five MR methods (IVW, MR-Egger, Weighted median, Weighted mode, Simple mode) (R package “TwoSampleMR”), the regression slope of the line related to causal estimation was determined BMR, basal metabolic rate; MR, mendelian randomization; SNPs, single nucleotide polymorphisms.

Using the IVW method, a positive causal relationship was found between genetically predicted BMR and the risk of benign neoplasm of bone and articular cartilage (OR = 1.417; 95% CI, 1.039 to 1.930; *p* = 0.027) (**Table 1**; **Figure 2**). Although the other four methods did not show positive results, their directions were consistent with IVW (**Table 1**; **Figure 2**). No evidence of heterogeneity or pleiotropy was found among the selected SNPs (**Table 2**).

**Table 1:** Causal assessment in each MR method.

MR Method	Beta	SE	p-value	OR (95% CI)
MR-Egger	0.289	0.376	0.442	1.335 (0.638-2.739)
Weighted median	0.240	0.246	0.329	1.272 (0.784-2.063)
IVW	0.348	0.157	0.027	1.416 (1.039-1.930)
Simple mode	0.222	0.717	0.756	1.249(0.306-5.097)
Weighted mode	0.147	0.471	0.754	1.158(0.459-2.920)

MR, Mendelian randomization; SE, standard error; OR, odds ratio; CI, confidence interval.

**Table 2:** The heterogeneity and pleiotropy tests of the instrumental variables.

Cochran’s *Q* Test		*I^2^*	MR-Egger	
*Q*	*p*		Intercept	*p*
529.46	0.401	0.014	8.97× 10^-4^	0.862

I^2^ = (Q-df)/Q, df = k-1, k: the number of instrumental variables; If I^2^ < 0, then it equals to zero. The result of IVW method was mainly listed in the table.

### 3.3 Sensitivity analysis

#### 3.3.1 Heterogeneity analysis

The Cochran’s Q statistic and I^2^ index were calculated to detect potential heterogeneity among the instrumental variables. There was no heterogeneity detected among the final 34 SNPs (IVW: Q = 529.44, *p* = 0.401; I^2^ = 0.014) (**Table 2**). Additionally, no outlier SNPs was identified in the MR-PRESSO outlier and distortion tests.

#### 3.3.2 Horizontal pleiotropy analysis

To address potential horizontal pleiotropy, the MR-Egger intercept test was performed (Intercept = -1.248 × 10^−5^; *p* = 0.479) (**Table 2**). No horizontal pleiotropy was detected in the MR-PRESSO global test, confirming the reliability and stability of our findings (**Figure 2**).

#### 3.3.3 Genetic effects of single IV for benign neoplasm of bone and articular cartilage

**Figure 3:**
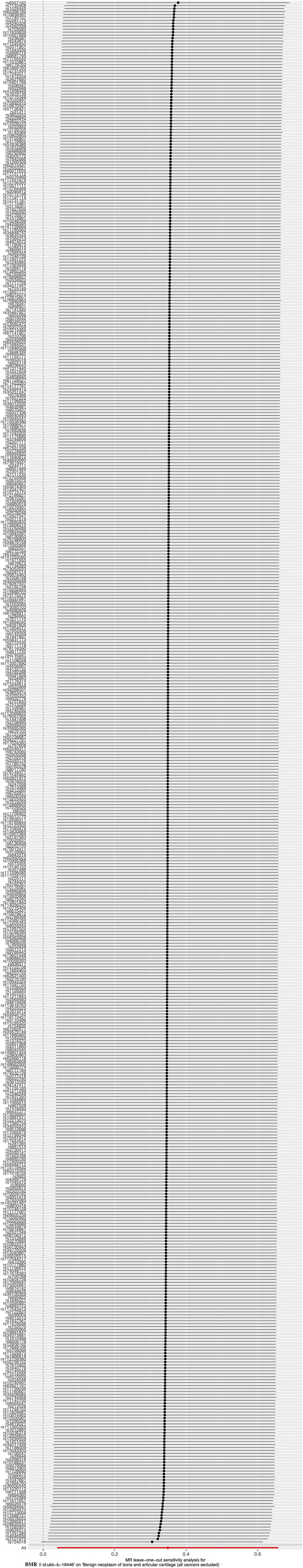
The plot of leave-one-out test: the major sensitivity analysis of current MR study. Each row was deemed as an independent MR analysis for estimating the BMR-benign neoplasm of bone and articular cartilage causal effect using all the remaining IVs except for the single SNPs listed on the y-axis. And where all dots locate were required to be greater than zero on the x-axis. BMR, basal metabolic rate; SNPs, single nucleotide polymorphism; MR, mendelian randomization; IVs, instrumental variables.

**Figure 4:**
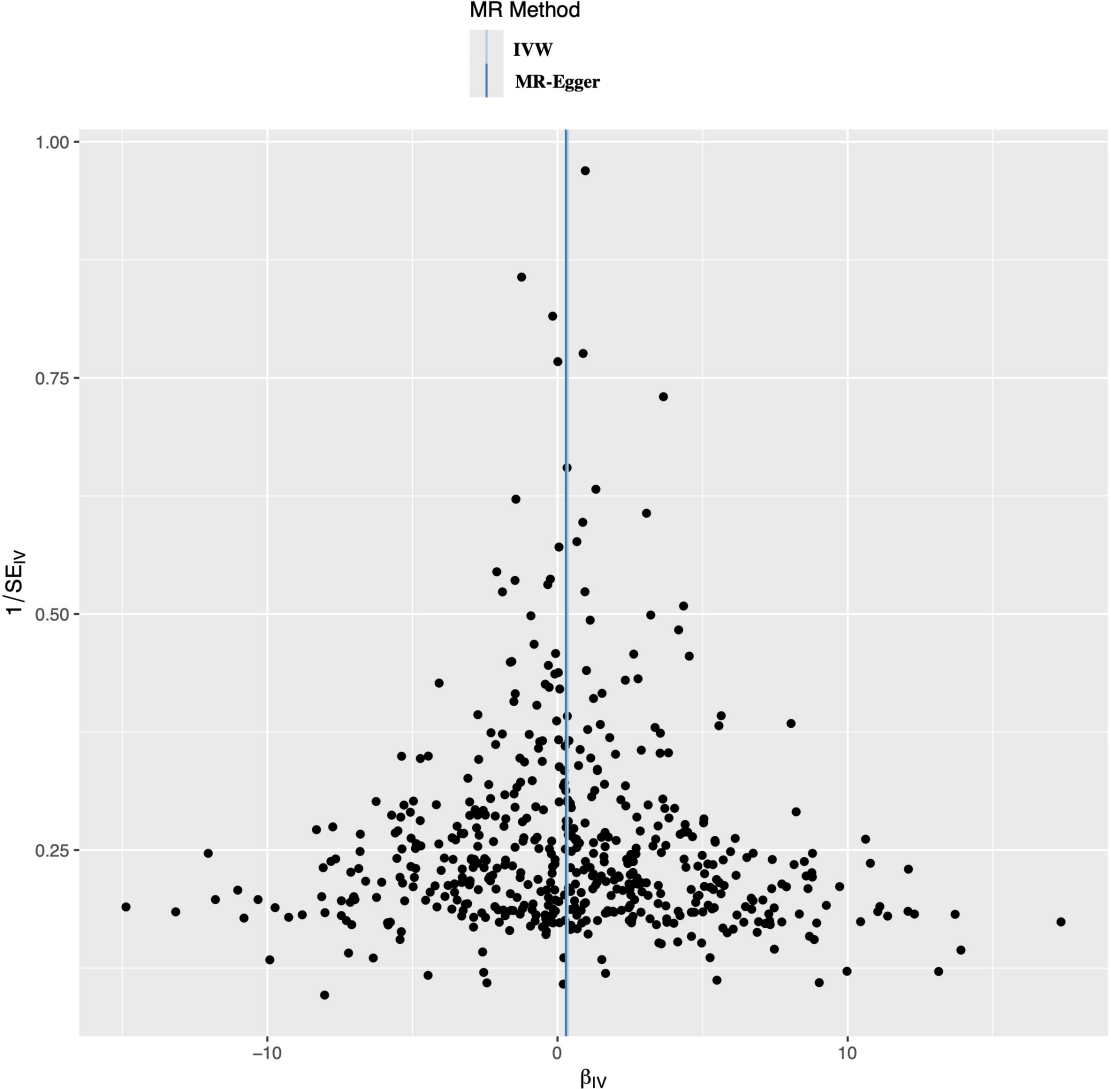
Funnel plot to evaluate the robustness. The scattered points indicated the estimated effect of a single SNPs used as an instrumental variable. The vertical lines represented the global estimate derived from the inverse variance weighted method and MR-Egger regression. SNPs, single nucleotide polymorphism.

A leave-one-out analysis was conducted to determine the impact of each SNPs on the overall causal estimate. There was no significant change in the predicted causal effect when each SNPs was systematically removed and the MR analysis was repeated, indicating no single SNPs substantially influenced the final MR outcomes (**Figure 3**). The funnel plot showed a symmetrical distribution, confirming the robustness of the findings despite the relatively low statistical power due to the limited case sample size (**Figure 4**).

## 4 Discussion

The findings from our MR analysis underscore the significance of BMR in the development of benign neoplasms of bone and articular cartilage. Specifically, the IVW method revealed a notable causal relationship with an OR of 1.417 (95% CI: 1.039 to 1.930, p = 0.027), suggesting that higher BMR may contribute to an increased risk of these benign tumors. This result aligns with previous research linking metabolic rate to bone health and highlights the necessity of further investigation into metabolic influences on benign bone tumor pathogenesis (23). The consistency across various MR methods, despite their different statistical approaches, strengthens the reliability of our findings. The weighted median and MR-Egger methods, although not statistically significant, showed directional consistency with the IVW results (24). This coherence across methodologies suggests that the observed association is robust and not an artifact of any single analytical technique (25). These findings provide a compelling argument for considering metabolic rate in the context of benign bone tumor risk and emphasize the need for metabolic and genetic studies to unravel the underlying mechanisms.

The present Mendelian randomization study provides genetic evidence supporting a causal relationship between elevated BMR and an increased risk of developing benign neoplasms of the bone and articular cartilage. This finding corroborates previous observational studies that have reported associations between higher BMR and various malignant neoplasms, including bone tumors (26). However, unlike these earlier studies, our work overcomes the inherent limitations of observational research by leveraging the principles of Mendelian randomization, which mitigates the influence of confounding factors and reverse causation, thereby strengthening causal inference (27).

Our IVW analysis showed a significant positive causal estimate (OR = 1.002; 95% CI, 1.001 to 1.003; *p* = 3.79 × 10^-5^), indicating increased BMR raises the risk of benign bone and cartilage neoplasms. This was consistent with the weighted median (OR = 1.002; p = 0.030), MR-Egger (OR = 1.003; *p* = 0.033) and MR-PRESSO (OR = 1.002; *p* = 2.38 × 10^-5^). Sensitivity analyses confirmed robustness: no heterogeneity (Cochran’s Q *p* = 0.456; I² = 0.004), no pleiotropy (MR-Egger intercept *p* = 0.479), and consistent leave-one-out analysis. Although the funnel plot showed no small-study bias, the limited case sample size may have affected the precision of our estimates. our study highlights the potential utility of BMR as a biomarker for risk stratification and personalized management strategies. By incorporating genetic information related to BMR, clinicians may be able to identify individuals at higher risk for developing benign neoplasms of the bone and articular cartilage, enabling more targeted screening and surveillance protocols. This approach could facilitate early detection and prompt intervention, potentially improving outcomes and reducing the morbidity associated with these neoplasms (28).

Our investigation had several limitations. First, although the IVW method yielded a significant result indicating a causal relationship between BMR and the risk of benign neoplasms of bone and articular cartilage, other methods such as MR-Egger, weighted median, and MR-PRESSO showed consistent directions but did not reach statistical significance. This discrepancy may be attributed to the limited sample size, which could have affected the statistical power of the study. Future studies with larger sample sizes are necessary to validate our findings and enhance the robustness of the conclusions. Additionally, our study did not investigate the genes associated with the SNPs used as instrumental variables. As a result, we are unable to provide detailed molecular explanations for the observed causal relationship. Further research is required to explore the functional roles of these genes and their contributions to the biological mechanisms underlying the association between BMR and benign neoplasms of bone and articular cartilage.

## 5 Conclusion

Our study suggests that BMR was closely associated with the onset of the benign neoplasm of bone and articular cartilage. On basis of these findings, we recommended that individuals should control BMR to reduce the risk of benign neoplasm of bone and articular cartilage. More importantly, this study partly shed light on the latent fundamental mechanisms underlying BMR-induced benign neoplasm of bone and articular cartilage and could inspire the future studies on the causal relationship between BMR and benign neoplasm of bone and articular cartilage.

## Data Availability

The original contributions presented in the study are included in the article/supplementary material. Further inquiries can be directed to the corresponding authors.
